# Translation, cross-cultural adaptation, and validation of the Duke Activity Status Index (DASI) to Sinhala language

**DOI:** 10.1186/s13741-024-00386-8

**Published:** 2024-05-13

**Authors:** C. Ranasinghe, K. Kariyawasam, J. Liyanage, Y. Walpita, U. Rajasinghe, A. Abayadeera, P. Chandrasinghe, M. Gunasekara, S. Kumarage, M. De Silva, K. Ranathunga, K. Deen, H. Ismail

**Affiliations:** 1 Center for Sport and Exercise Medicine, Faculty of Medicine, University of Colombo, Colombo, Sri Lanka; 2 Department of Allied Health Sciences, Faculty of Medicine, University of Colombo, Colombo, Sri Lanka; 3 Department of Community Medicine, Faculty of Medicine, University of Colombo, Colombo, Sri Lanka; 4 Department of Anaesthesiology, Faculty of Medicine, University of Colombo, Colombo, Sri Lanka; 5 Department of Surgery, Faculty of Medicine, University of Kelaniya, Colombo, Sri Lanka; 6 Department of Cardiothoracic Anaesthesiology, National Hospital of Sri Lanka, Colombo, Sri Lanka; 7 Cardio Pulmonary Exercise Testing (CPET) Laboratory, Peter MacCallum Cancer Hospital Melbourne, Melbourne, Australia

**Keywords:** DASI, Pre-operative, Physical activity, Sri Lanka, Sinhala

## Abstract

**Background:**

Duke Activity Status Index (DASI) is a widely used tool to assess functional capacity among patients, but there is no Sinhala version validated for patients in Sri Lanka. This study aimed to cross-culturally adapt and test the validity and reliability of the Sinhala version of DASI (DASI-S).

**Methods:**

The translation and cross-cultural adaptation of the DASI questionnaire were conducted following the standard guidelines. It was pre-tested on ten pre-operative patients and further modified. The construct validity and reliability of DASI-S were evaluated by administering the modified final DASI-S, which comprised 12 items, along with the physical functioning sub-scale of the 36-item short-form health survey (SF-36), consisting of 10 items to eighty-one patients who were awaiting non-cardiac surgeries at university surgical wards, National Hospital of Sri Lanka (NHSL), and Colombo North Teaching Hospital (CNTH), Sri Lanka. Reliability was assessed through Cronbach alpha, while the validity was evaluated using factor analysis and Spearman’s correlation. The ethical approval was obtained from the Ethics Review Committee, Faculty of Medicine, University of Colombo, Sri Lanka.

**Results:**

The mean age of the participants was 46.2 (± 16.6) years and the majority were females (54.3%). The mean height, weight, and body mass index of the sample were 160.5 (± 9.6) cm, 60.3 (± 11.9) kg, and 23.4 (± 4.5) kgm^−2^ respectively. The Cronbach's alpha coefficient for the internal consistency of DASI-S was 0.861. The concurrent validity of DASI-S was substantiated by positively correlating (*p* < 0.01, *r*_s_ = 0.466) with the physical sub-scale of SF-36. There was a significant difference (*p* < 0.01) in the total score of DASI-S between the two age groups.

**Conclusions:**

Sinhala version of the DASI appears to be a valid, reliable and easy-to-administer tool to assess functional capacity among patients who are awaiting non-cardiac surgeries.

## Background

Surgery is required to treat one-third of the global disease burden, and two-thirds of patients with cancer. After ischemic heart disease and stroke, post-operative deaths account for the third largest proportion of all deaths worldwide (Nepogodiev et al. 2019) and are primarily driven by postoperative complications (Khuri et al. 2005), occurring in one in every four patients (Duckett and Jorm 2018).

The number of major surgeries carried out annually worldwide amounts to 234.2 million (Lai and Hosie 2012). It has been identified that advancing age and co-morbidities increase healthcare utilization for surgical purposes (Palladino et al. 2016, Park et al. 2013). These factors lead to increased post-operative complications as well as increased length of hospital stay (Palladino et al. 2016). An optimum pre-operative assessment is important to identify risk factors to enable the implementation of evidence-based strategies and interventions aimed at reducing complications following major surgery. Deconditioning and co-morbidities can lead to impaired cardiorespiratory fitness and reductions in functional capacity. These factors are recognized as predictors of post-operative morbidity and mortality, as evidenced by the Measurement of Exercise Tolerance before Surgery [METS] study which presented a protocol to measure exercise tolerance before surgery which is important for identifying risk (Lai and Hosie 2012, Wijeysundera et al. 2016). Accurate pre-operative assessment of the functional capacity is therefore crucial to improving surgical outcomes by optimizing modifiable risk factors (Park et al. 2013).

Cardiopulmonary exercise testing (CPET) is the gold standard objective assessment of functional capacity and cardiorespiratory fitness (Costa et al. 2014, Levett et al. 2018). However, CPET is resource-intensive, costly, and time-consuming and is not accessible to all pre-operative patients; especially in resource-poor settings (Lai and Hosie 2012, Neves et al. 2013). The Duke Activity Status Index (DASI) is a fast, simple, and cost-effective tool initially developed to measure the functional capacity of patients with cardiovascular diseases (Hlatky et al. 1989) and is now widely used in pre-operative patients to predict functional capacity (El-Kefraoui et al. 2021, Riedel et al. 2021) and post-operative complications (Wijeysundera et al. 2016). The DASI consists of 12 items addressing self-care, ambulation, household chores, sexual activity, and recreational activities. Each item is scored proportionally to the metabolic cost of each activity in metabolic equivalents (METs). It specifically assesses an individual’s ability to perform activities of daily living and they are scored accordingly. Since DASI is used as a self-administered questionnaire, it needs to be administered in an appropriate language for better understanding and application. Up to now, it has been translated into Portuguese, Hindi, Turkish, Brazilian Portuguese, and Thai languages (Neves et al. 2013, Coutinho-Myrrha et al. 2014; Govil et al. 2020, Mustafaoglu et al. 2021).

The DASI can be used to assess functional capacity in a Sri Lankan context. However, it has not been translated and cross-culturally adapted to Sinhala, the native majority language of the country.

The objective of this study was to develop the Sinhala version of the Duke Activity Status Index (DASI-S), cross-culturally adapt, and evaluate the reliability and validity of DASI-S for the evaluation of functional capacity among pre-operative patients in Sri Lanka.

## Methods

### Phase I—Translation and cross-cultural adaptation

#### Translation

Translating the original DASI questionnaire into the Sinhala language was conducted based on the guidelines introduced by Beaton and colleagues (Beaton et al. 1976). The original English version of DASI was independently forward-translated into Sinhala by two qualified, bilingual, native Sinhala-speaking translators. Translation 1 (T1) and Translation 2 (T2) versions were generated by them. Both translators independently forwarded and translated the questionnaire and made a report about doubts and difficulties faced during the translation process. Then a common Sinhala translated version (T3) was created using the original questionnaire and the first and second translator’s Sinhala versions (T1, T2).

The T3 version was back-translated independently into English, by two qualified native English-speaking translators. They were not informed about the objectives of the study and had no access to the original version. Neither of these translators were from a medical background. The back-translated version 1 and version 2 (BT1 and BT2) from the above-mentioned English-speaking translators were then compared with the original version by the review committee. Back translation is only one type of validity check, highlighting main inconsistencies or conceptual errors in the translation.

#### Cross-cultural adaptation

This phase was conducted using the Delphi technique which is a widely used and accepted method for gathering data from respondents within their domain of expertise (Boulkedid et al. 2011).

#### Expert committee review

An expert committee consisted of translators, healthcare professionals, and an expert in research methodology all of whom understood the concepts and goals of DASI. The committee assessed the clarity, relevance, consistency, and significance of the items based on the socio-cultural context of Sri Lanka. The experts were requested to review each item in the questionnaire and to indicate whether the item should be retained in the questionnaire to assess the functional capacity of non-cardiac pre-operative patients in Sri Lanka. If they decided that the item should be retained, then they were asked to assess the cultural appropriateness of the words and examples used in the items on a 1–5 scale. If they were assigned a score less than 3, they were further asked to indicate their suggestion on how the item should be modified to improve the cultural appropriateness. Items 3, 7, 11, and 12 had mean scores below 4. Hence, they were modified (Table 1). For instance, in item 3, it was noted that “blocks” were not a standard unit of measurement of distance in Sri Lanka. In item 7, “vacuuming” and in items 11 and 12 “golf”, “bowling” and “skiing” are not activities commonly indulged in in Sri Lanka. They were replaced with equivalent activities for metabolic expenditure. All items of the pre-final version were evaluated and compared to the original version to achieve semantic, idiomatic, conceptual, and content equivalence. The pre-final version was approved by all the members of the committee, stating that all items were clear and would be easily understood by the equivalent of a 12-year-old (Beaton et al. 1976).

**Table 1:** Modified items of DASI-S version

Item no.	Original version	Modified (SL) version
03	Walk a block or 2 on level ground?	Walk 50–100 m on level ground?
07	Do moderate work around the house like vacuuming, scrubbing the floor or carrying in groceries?	Do moderate work around the house like sweeping, scrubbing the floor or carrying in groceries?
11	Participate in moderate and recreational activities like golf, bowling, dancing, doubles tennis or throwing a baseball or football	Participate in moderate and recreational activities like fast walking, slow cycling, dancing, throwing a cricket or football, doubles tennis, netball
12	Participate in strenuous sports like swimming, singles tennis, football, basketball, or skiing?	Participate in strenuous sports like running, swimming, football, basketball, singles tennis, or badminton

#### Pre-test

The pre-final version was administered to 10 pre-operative participants to evaluate possible deviations and errors committed during translation. Individuals were tasked with reading and responding to each item to assess whether a clear understanding of the questionnaire alone enabled the respondent to answer accurately, or if other related factors played a role. All participants of the pre-test stated that the items were clear; easy to answer; knew all the activities listed and they had no doubts during application.

#### Sample size

Sample size was calculated considering the recommendations for the ratio of respondents to items varied, ranging from 5:1 (e.g., 50 respondents for a 10-item questionnaire) to 10:1 (Tsang et al. 2017). Since DASI has 12 items 60 to 120 participants was the range. Eighty-one participants were recruited for the study.

### Phase II—validation design, sample, and setting

A cross-sectional study was conducted to assess the reliability and validity of the DASI-S in a group of individuals who were awaiting non-cardiac surgeries. This study received approval from the Ethics Review Committee, Faculty of Medicine, University of Colombo, under reference number EC-22-050. Data was collected from May 2023 to October 2023 by two trained research assistants. The study was conducted at the University Surgical wards, National Hospital of Sri Lanka (NHSL), and Colombo North Teaching Hospital (CNTH) Sri Lanka. A sample of 81 patients was recruited after obtaining the written informed consent to participate in the study.

### Measurements

In this study, two instruments were used: DASI-S and the physical functioning sub-scale of 36-item short-form health survey (SF-36) (Vibulchai et al. 2014).

### DASI-S

DASI-S evaluated the functional capacity in the areas of personal care, ambulation, household tasks, sexual function, and recreation (Hlatky et al. 1989). Each participant within the patient cohort was asked about their ability to conduct various activities. The response modality employed was binary, denoted as “yes” or “no”. In instances where a participant answered “Yes,” the activity received a score predicated on the established metabolic expenditure associated with each activity (Hlatky et al. 1989). The assigned scores for the activities fell within the range of 1.75 to 8.0. Conversely, if a marked “no”, the score for that activity was zero. The cumulative score, indicative of functional capacity, exhibited a potential range from 0 to 58.2, wherein a score of 0 denoted the poorest functional capacity, and a score of 58.2 denoted the highest functional capacity. Higher cumulative scores were indicative of higher functional capacities (Hlatky et al. 1989).

This cumulative score was used to estimate oxygen consumption (VO_2)_ (ml×kg^−1^×min^−1^), which is the rate (V) of oxygen (O_2_) the body is able to use during exercise/activity. This is an objective measure of cardiorespiratory fitness and functional capacity and can be predicted using the following equation (Riedel et al. 2021) as per the findings of the original DASI questionnaire validation which is expected to hold true in any population.

$$VO2 = 0.43 \times DASI + 9.6$$

### SF-36

The Sinhala-translated version of the physical functioning sub-scale of SF-36, which is a 10-item questionnaire in version 2.0 was also administered to the same patients. The SF-36 was translated and cross-culturally adapted into the Sinhala language (Gunawardena et al. 2003). It measures the perceived limitations of an individual, related to physical activities due to health issues (Brazier et al. 1992). Respondents indicate if their health restricts physical activity, basic mobility, and daily living tasks, rating their current limitations on a 3-point Likert scale (yes, limited a lot; yes, limited a little; and no, not limited at all). To score and interpret the results, weighted responses to all questions are totalled to create raw scores, which are then transformed to a 0–100 scale (Ware and Sherbourne 1992, Ware 1976). Higher scores interpret more favourable health status.

### Statistical analysis

Statistical analyses were conducted using the Statistical Package for the Social Sciences version 23. Descriptive statistics are presented as percentages, mean plus or minus (±) standard deviation (SD), while ceiling and floor effects were determined by assessing the percentage of participants attaining the maximum and minimum achievable scores. Cronbach’s alpha was employed to evaluate internal consistency.

Construct validity of the instrument was appraised through the convergent validity between the results of DASI-S and the physical sub-scale of SF-36 using Spearman’s rank correlation coefficient and by conducting a factor analysis. Factor structure was explored through principal components analysis with varimax rotation with Kaiser normalization. The combination of Varimax rotation with Kaiser Normalization is often used in exploratory factor analysis to produce a set of factors that are uncorrelated and easier to interpret, while also accounting for differences in variance among factors. This method is widely used due to its simplicity and effectiveness in revealing the underlying structure of a set of variables. The adequacy of the correlation matrix was verified by the Kaiser-Meyer-Olkin criteria, which should be greater than 0.60 and Bartlett's test considering a significance level of 0.05. Eigenvalues greater than or equal to one were considered to extract the relevant factors. Following the rotation matrix, items with a factor loading greater than or equal to 0.4 were added to the factor. To assess known-group validity, the independent sample Mann-Whitney *U* test was used to compare DASI-S total scores by age group (age < 50 years, age ≥ 50 years). All statistical tests were performed at the two-tailed 5% level of significance and Spearman’s correlation coefficient ± 0.3 to ± 0.5 interpreted as fairly correlated (Akoglu 2018).

## Results

### Description of the sample and scores on measures

Eighty-one subjects were enrolled for the validation study from the National Hospital of Sri Lanka/NHSL (66.7%) and Colombo North Teaching Hospital/CNTH (32.3%). The mean age of the participants was 46.2 (± 16.6) years and 53.1% were below 50 years. The mean height, weight, and body mass index of the sample were 160.5 (± 9.6) cm, 60.3 (± 11.9) kg, and 23.4 (± 4.5) kgm^−2^ respectively. The socio-demographic characteristics of these participants are presented in Table 2; total scores of DASI and SF-36 and VO_2_ of the sample are presented in Table 3.

**Table 2:** Socio-demographic characteristics of the participants

Socio-demographic characteristics (*N* = 81)	Frequency	Percentage %
Gender		
Male	37	45.7
Female	44	54.3
Civil status		
Married	60	74.1
Unmarried	17	21.0
Widowed	2	2.5
Divorced/separated	2	2.5
Current employment status		
Unemployed	41	50.6
Self-employed	4	4.9
Retired	10	12.3
Employed	26	32.1
Educational qualifications		
Not up to ordinary level	2	2.5
Up to ordinary level	41	50.6
Up to advanced level	21	25.9
Higher diploma/vocational training	3	3.7
Bachelor’s degree	12	14.8
Post-graduate	2	2.5

The scores obtained by the patients for the DASI-S, SF-36, and the calculated VO_2_ from the DASI score are stated in the table below.

**Table 3:** Total scores of DASI and SF-36 and VO_2_ of the sample

	Minimum	Maximum	Mean	SD
DASI-S	2.70	58.20	43.13	16.32
SF-36	0.00	1000.00	645.06	282.69
VO_2_	10.76	34.63	28.15	7.02

### Reliability

Cronbach’s α coefficient for the DASI-S was 0.861, indicating a high reliability. There was no floor effect on both DASI-S and physical sub-scale of SF-36, but a ceiling effect was observed in DASI-S (33.33%).

### Validity

#### Concurrent validity

**Fig. 1:**
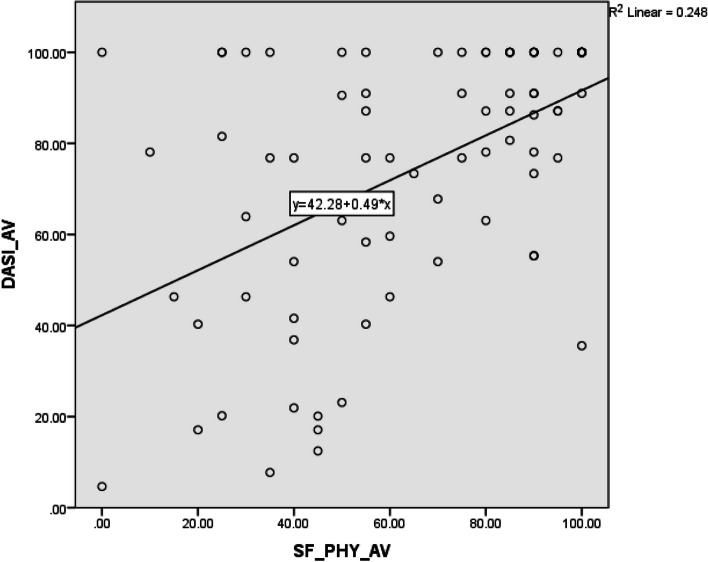
Correlation between average scores of DASI and physical fitness sub-scale of SF-36 questionnaires

The average score of DASI-S was positively correlated (*p* < 0.01, *r*_s_ = 0.466) (Fig. 1) with the physical sub-scale of SF-36 average score, confirming the correlation of DASI with a similar construct.

#### Factor analysis

**Fig. 2:**
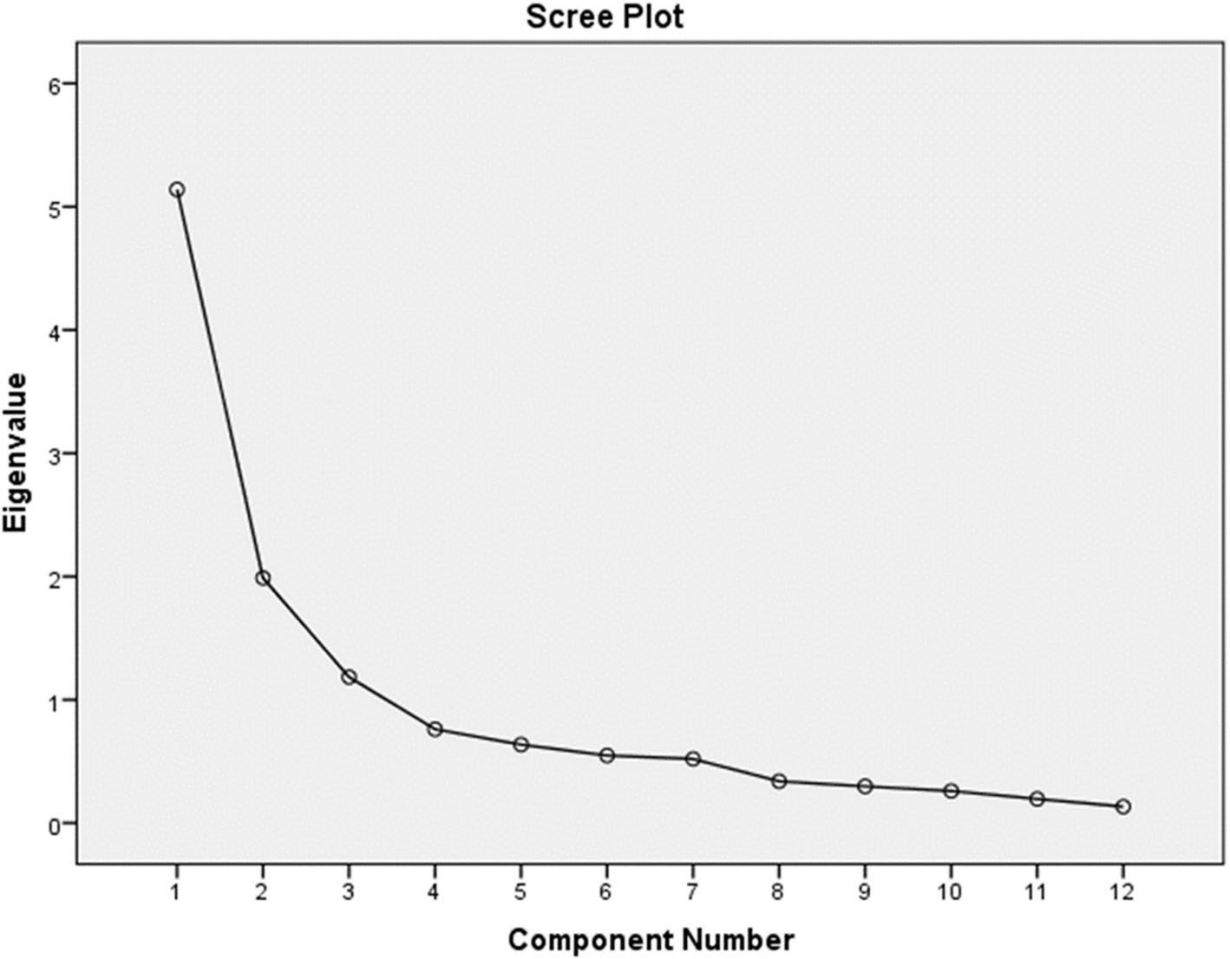
Scree plot of DASI-S

The factor structure of the DASI-S was explored through principal component analysis via Varimax rotation with Kaiser Normalization. Rotation converged in 5 iterations. The 12 items of the DASI converged into three distinct factors with items depicting mild (D1,2,3), moderate (D6,5,7,4) and high (D11,12,8,9,10) metabolic demand grouping together respectively. The total variance explained by the three-factor model described above was 69.75%. The Kaiser-Meyer-Olkin value was 0.783 while Bartlett's Test of Sphericity was significant (Fig. 2).

The scree plot also indicates that three factors had eigenvalues of more than one.

#### Known group validity

There was a significant difference (*p* < 0.01) in the total score of DASI-S between the two age groups (Table 4), which means the DASI-S is able to differentiate between patients in different age groups. The DASI total score for patients aged < 50 years was significantly higher than the patients aged ≥ 50 years.

**Table 4:** DASI total score for patients according to the age groups

Group	Mean ± SD	*p*
< 50 years (*n* = 43)	49.09(13.88)	0.000
≥ 50 years (*n* = 38)	35.63(16.84)	

## Discussion

This research aimed to translate, cross-cultural adapt the DASI to Sinhala language (DASI-S) and validate the suitability of the DASI-S for assessing the functional status of Sri Lankan patients who are awaiting non-cardiac surgeries. The validation process involved a systematic approach. We reviewed established instruments for measuring the functional status of patients who are waiting for non-cardiac surgeries and selected relevant tools. Following the International Society for Pharmacoeconomics and Outcomes Research task force's translation guidelines, a cross-cultural investigation was conducted, employing a team approach with independent bilingual translators to identify and address issues related to translated measures, including vocabulary, experiential, and conceptual equivalence. The translation process ensured content equivalence between the DASI-S and its original version.

### Reliability

Internal consistency, the extent to which items in a questionnaire are correlated, thus measuring the same concept was determined by assessing the average correlation between items within the test (Tsang et al. 2017). According to the literature, a new instrument with an internal consistency of 0.65–0.80 is often considered “adequate” for a scale used in human dimensions research (Vaske et al. 2017). In our study, the internal consistency of the DASI-S, measured by Cronbach’s α coefficient, was deemed acceptable at 0.861. A high α value is indicative that the items effectively measure an underlying construct (Vaske et al. 2017). The internal consistency outcome in this research aligns with a prior study demonstrating a notably elevated Cronbach's α coefficient, similar to the earlier findings (Coutinho-Myrrha et al. 2014, Vibulchai et al. 2014; Alonso et al. 1997).

A ceiling effect was only observed for the DASI-S total score. These results are consistent with a previous study which was done in Spain (Alonso et al. 1997). They have also identified a ceiling effect of 34.8 which is almost similar to our finding. These findings provide evidence supporting the reliability of the DASI-S as a tool for measuring functional status in patients who are awaiting non-cardiac surgeries.

### Validity

Satisfactory construct validity was confirmed with the concurrent validity observed in the correlation between the DASI-S and the SF-36 physical functioning sub-scale. A notably fair correlation emerged between the DASI-S total score and the SF-36 physical functioning sub-scale total score (*r* = + 0.466, *P* < 0.01). The principal component analysis revealed meaningful factor structure depicting mild, moderate, and high physical activity level items grouped together. The DASI-S also demonstrated known-group validity; a subtype of construct validity evident when there is a significant difference between the pre-identified groups. In the known-group validity testing of DASI-S, total scores across age groups were significantly different, indicating that the DASI-S is a valid measure for discerning diverse scores in various clinical characteristics associated with the functional status of samples. These align with findings from recent DASI validation studies such as Thai and Portuguese versions (Coutinho-Myrrha et al. 2014, Vibulchai et al. 2014). Collectively, this evidence substantiates the validity of the DASI-S as an assessment tool for determining the functional status of patients who are awaiting non-cardiac surgeries.

This study had a few limitations. The analysis was restricted to a non-normal distribution of scores.

Also, although we used Cronbach’s α, it has its limitations: it assumes that all test items are equally reliable, which may not be the case.

Furthermore, the sample size was small and the illiterate individuals who could not read and write Sinhala were excluded. Further studies are required to clarify these aspects.

## Conclusion

The current adaptation of the DASI for the Sinhala language is tailored to Sri Lankan culture, demonstrating apparent validity, reliability, efficiency, and simplicity as an instrument for evaluating and risk-stratifying the functional capability of individuals who are awaiting non-cardiac surgery. This Sri Lankan version (DASI-S) can be used in clinical practice and in research to benchmark and compare Sri Lankan studies with those from other countries using the same tool. This study also provides evidence that translation to other native languages of Sri Lanka (Tamil) is feasible and necessary.

## Data Availability

The data that support the findings of this study are not openly available due to reasons of sensitivity and are available from the corresponding author upon reasonable request. The data is securely stored in controlled access storage at the Faculty of Medicine, University of Colombo.

## Abbreviations

CPET: Cardiopulmonary exercise testing
CNTH: Colombo North Teaching Hospital
DASI: Duke Activity Status Index
DASI-S: Sri Lankan version of Duke Activity Status Index
METs: Metabolic equivalents
NHSL: National Hospital of Sri Lanka
SD: Standard deviation
SF-36: Short-Form Health Survey
VO_2_: Oxygen consumption
